# Protein Markers for the Differential Diagnosis of Vascular Dementia and Alzheimer's Disease

**DOI:** 10.1155/2012/824024

**Published:** 2012-06-04

**Authors:** A. H. Simonsen, N.-O. Hagnelius, G. Waldemar, T. K. Nilsson, J. McGuire

**Affiliations:** ^1^Memory Disorders Research Group Section 6702, Department of Neurology, Copenhagen University Hospital Rigshospitalet, Blegdamsvej 9, 2100 Copenhagen, Denmark; ^2^Department of Geriatric Medicine, Örebro University Hospital, 701 85 Örebro, Sweden; ^3^Department of Laboratory Chemistry, Örebro University Hospital, 701 85 Örebro, Sweden; ^4^Department of Incretin Biology, Novo Nordisk, Niels Steensens vej 1, 2820 Gentofte, Denmark

## Abstract

Alzheimer's disease (AD) is the most common form of dementia found in all human populations worldwide, while vascular dementia (VaD) is the second most common form of dementia. New biomarkers for early and specific diagnosis of AD and VaD are needed to achieve greater insight into changes occurring in the brain and direct therapeutic strategies. The objective of this explorative study was to discover candidate protein biomarkers for the differential diagnosis between VaD and AD. Surface-enhanced laser desorption/ionization (SELDI) TOF-MS was used to differentially profile proteins and peptides in CSF samples from 28 AD patients and 21 patients with VaD. A combination of univariate (Kruskal-Wallis) and multivariate (independent component analysis) statistical approaches produced a list of 27 proteins and peptides that could differentiate between VaD and AD. These markers represent various physiological processes, such as protein degradation (ubiquitin), protease inhibition (cystatin C and alpha-1-antichymoptrypsin), and inflammation (C3a and C4a) that are known to be represented in neurodegenerative diseases.

## 1. Introduction

Alzheimer's disease (AD) is one of the most devastating brain disorders in the elderly. AD is a progressive neurodegenerative disease that represents the most common form of dementia today [1]. Age is the single most prominent risk factor with the incidence doubling every five years from the age of 65 [2].

Vascular dementia (VaD) is a heterogeneous disorder that accounts for about 20% of all cases of dementia. Vascular dementia is characterized by neuronal death due to vascular lesions such as lacunar, cortical or subcortical infarcts, cerebral hemorrhage, and cardiogenic embolism, contributing to cognitive decline [3].

Vascular dementia and Alzheimer's disease frequently occur together and they may often act in combination to cause dementia and it is clinically challenging to separate these two diseases.

Biomarkers that can aid in the differential diagnosis between VaD and AD are relevant for several reasons. First, the pharmacological treatment strategy differs between the two diseases since patients with VaD only benefit modestly if at all from cholinesterase inhibitors and memantine which are the drugs used to treat AD. Since there are no approved drugs for the treatment of VaD, current treatment is limited to the control of known vascular risk factors, such as hypertension and dyslipidemia [4]. Second, with new AD disease modifying drugs being tested it is crucial to differentiate between the patient groups for trial selection. Third, from an epidemiologic point of view it is of utmost importance that the clinical classification is based on the most up-to-date diagnostic practices in order to select the most appropriate predictor variables and monitoring tolls.

The aim of this explorative study was to discover candidate protein biomarkers for the differential diagnosis of VaD and AD.

## 2. Materials and Methods

### 2.1. Study Subjects and Samples

Patients for this study were recruited at University Hospital, Örebro, Sweden. They were all referred to the Memory Care Unit at the Department of Geriatrics for diagnostic assessment and treatment of suspected cognitive problems. According to Swedish research ethics law no proxy could sign the informed consent papers on behalf of the patient when collecting biobank material. All patients in the study group underwent a structured and thorough clinical investigation, including medical history, family history and socioeconomic data, and physical as well as neurological and psychiatric examination.

Considering the aim of this study, an important goal was to include cases with as narrowly defined diagnoses as possible in order to exclude cases with possible mixed AD/VaD, which would otherwise attenuate the differences between the two groups.

Blood chemistry tests were done on all (see below). Cerebrospinal fluid (CSF) samples were obtained by lumbar puncture at L3/L4 or L4/L5 level. The first 10–12 mL were collected in polypropylene tubes. The lumbar puncture and blood samples were collected at the same time, between 08 and 10 AM after overnight fast. The samples were centrifuged and supernatants stored in a biobank freezer at −80°C.

The presence or absence of dementia was diagnosed according to Diagnostic and Statistical Manual of Mental Disorders (DSM-IV) criteria. Probable AD was diagnosed according to National Institute of Neurological and Communicative Diseases and Alzheimer's Disease and Related Disorders Association (NINCDS-ADRDA) criteria [5], VaD was diagnosed according to National Institute of Neurological Disorders and Stroke and Association Internationale pour la Recherché et l'Enseignement en Neurosciences (NINDS-AIREN) criteria [3]. Disease severity was assessed using MMSE scores [6].

CSF samples (10–12 mL) were obtained by lumbar puncture, collected in polypropylene tubes, and gently mixed. The samples were centrifuged at 2000 × g for 10 min to remove cells and other insoluble material. Supernatants were frozen in aliquots and stored at −80°C. To avoid blood contamination, samples were discarded if they contained more than 500 erythrocytes per *μ*L.

The study was approved by the Regional Ethical Review Board, Uppsala. All patients gave informed consent to participate in the study, which was conducted in accordance with the provisions of the Helsinki Declaration.

### 2.2. Laboratory Methods

CSF samples were thawed and 5 *μ*L of each sample was diluted into 45 *μ*L of the appropriate binding buffer for each of the ProteinChip Array types (Ciphergen Biosystems, Fremont, CA, USA) using surface-enhanced laser desorption/ionization time-of-flight mass spectrometry (SELDI-TOF-MS). The array types used were CM10 and IMAC30 coupled with nickel and H50. To ensure reproducibility of sample preparation and array analysis, a reference CSF standard was randomly distributed in several separate aliquots among the clinical samples and analyzed under exactly the same conditions. Reproducibility was measured by calculating average coefficients of variation (CV) for each set of acquisition parameters. All-array preparation was performed using a Biomek 2000 robot (Beckman Coulter) and randomized sample placement. The samples were allowed to bind for 60 minutes at room temperature. Each array was washed three times with the appropriate binding buffer and rinsed twice with water. Energy absorbing molecule (EAM) application was performed automatically using a modified Biodot AD3200 robot. Two aliquots of 0.75 *μ*L of solution containing 12.5 mg/mL sinapinic acid (SPA) in 50% acetonitrile, 0.5% trifluoroacetic acid (TFA) were applied with drying in a controlled atmosphere between applications. The arrays were read at two different instrument settings to focus on lower and higher masses. Each sample was run in duplicates on separate arrays on successive robot runs. All arrays were analyzed using a SELDI-TOF MS ProteinChip Reader, series PCS4000 (Ciphergen Biosystems, Fremont, CA, USA). A protein profile was generated in which individual proteins were displayed within spectra as unique peaks based on their mass-to-charge ratio (m/z). Intensity normalization was done by calculating the total ion current for a spectrum, and the total ion current was then divided by the number of data points for that spectrum to obtain an average ion current for each individual spectrum. An external coefficient of 0.2 was chosen against which each spectrum was normalized. The very low mass region contains chemical “noise” from the matrix and was therefore excluded from the analysis.

### 2.3. Purification and Identification of Candidate Markers

Biomarkers were purified using combinations of chromatographic techniques employing a range of sorbents (Pall corporation, NY, USA) typically followed by SDS-PAGE. One-quarter of a gel band was extracted using a solution containing 50% formic acid, 25% acetonitrile, 15% isopropanol, and 10% water and reanalyzed using the ProteinChip Reader to confirm their exact masses matched with the original biomarker. The remaining three-quarters of the band were in-gel digested with trypsin. Alternatively, whole bands of interest were extracted from gels and reanalyzed using the ProteinChip Reader to confirm their exact masses matched with the original biomarker. The gel-extracted proteins were in-solution digested with trypsin. Tryptic digests were analyzed by peptide mapping using the ProteinChip Reader and by MS/MS using a Q-STAR XL tandem mass spectrometer (AB Sciex) fitted with a PCI-1000 ProteinChip Interface (Ciphergen Biosystems, Fremont, CA, USA). Biomarkers smaller than 4 kDa were enriched by combinations of chromatographic techniques and identified directly by tandem MS without SDS-PAGE purification and/or trypsin digestion.

Peptide mass fingerprints and amino acid sequencing results were entered into the Mascot database (http://www.matrixscience.com) for protein identification.

### 2.4. Bioinformatics and Statistical Methods

ProteinChip profiling spectral data were collected using CiphergenExpress data management software version 3.0 (Ciphergen Biosystems, Fremont, CA), where data handling and univariate analysis were also performed. All spectra were internally mass calibrated and peak intensities were normalized using total ion current.

All statistical analyses were conducted in R (http://www.r-project.org/). The data structure complexity and redundancy were reduced by identifying peak families (Spearman correlation coefficient ≥0.85) and retaining only the most intense member. Comparisons were performed using a Mann-Whitney test with a Benjamini-Hochberg (BH) correction for multiple testing. A signal processing algorithm, independent components analysis (ICA), was applied to the data set in addition to the two group comparison [7].

ICA decomposes the data matrix (E) into a component matrix (C) comprised of the independent components (ICs) and a mixing matrix (A) giving the linear mixing of each IC in the samples. It is written as E = CA. An entry in the component matrix is denoted a load and for each IC the loads have a mean of zero and a standard deviation of one. The fastICA package [8] was used to decompose data by applying ICA and estimate the ICs. ICA was applied on data a total of five times and the results from each run were tested for stability by calculating an absolute correlation coefficient between the loads in each of the ICs with a set of ICs obtained from 100 independent runs of fastICA [9]. In each of the 100 runs, the maximum absolute correlation coefficient was saved and only components with a mean absolute correlation coefficient ≥0.80 were retained. The capability of each IC to separate the samples according to classification was tested using a Mann-Whitney test with Benjamini-Hochberg correction for multiple testing. Peaks from significant components (adjusted *P* value <0.05) having absolute loads greater than 5 times the standard deviation of the absolute loads were extracted as candidate peptide biomarkers.

## 3. Results

In this study we analyzed CSF samples from 28 patients with AD and 21 patients with VaD (see Table 1 for age, gender, and MMSE).

Neither age nor MMSE was significantly different between the AD and VaD groups with *P* values of 0.71 and 0.28 for age and MMSE, respectively.

As expected, levels of Amyloid Beta 1–42 were significantly decreased in patients with AD and levels of Total Tau and Phosphorylated Tau were significantly increased in the same patient group compared to patients with VaD.

The albumin ratio was significantly higher in patients with VaD and the folate ratio was significantly lower for the same patient group as described before [10].

The SELDI intra-assay reproducibility of the discovery method was measured on median peak intensities in 48 individual spectra from a reference CSF sample and found to be between 14 and 19 percent CV (data not shown).

In Table 2 we describe the number of peaks found on the three SELDI array surfaces as well as the number of significant peaks. The CM10 surface had considerably more peaks, as well as significant peaks, which are summarized in Tables 3(a) and 3(b).

In total, eleven candidate biomarkers were obtained by a two-group comparison (Table 3(a)). Independent component analysis (ICA) gave rise to 21 significant components (Table 3(b)). There were six peaks in common between the two methods. ICA has the power to extract single variables or small groups of variables from a complex data set. The components can be analyzed for a particular characteristic, such as the ability to separate the two biological groups. The combination of this method with a classical univariate approach yielded a significant number of proteins to consider for further studies.

Unique peaks were found by analysis with ICA and Mann-Whitney, which emphasizes the complementary effect of combining feature reduction approaches.

**Figure 1:**
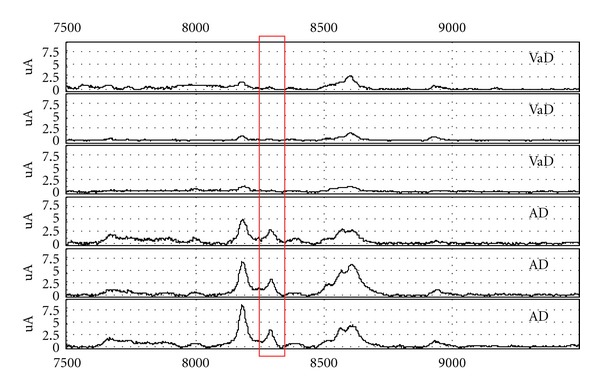
Representative spectra of Ubiquitin −3aa from CT on CM10 array.

**Figure 2:**
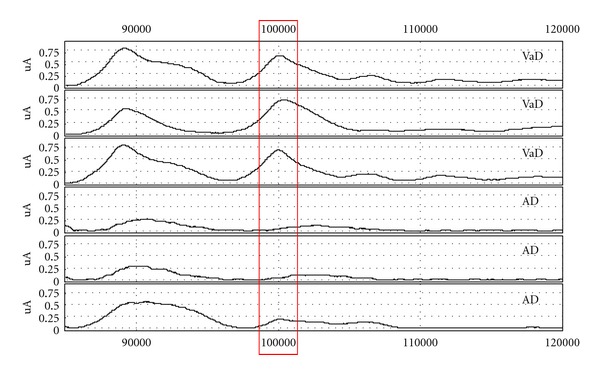
Representative spectra of 101 kDa marker on CM10 array.

**Figure 3:**
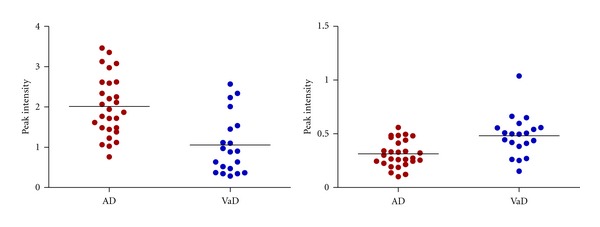
Scatter plot of peak intensities of Ubiquitin lacking 3 amino acids at the C-terminus left panel and the 101 kDa marker right panel.

We found a total of 27 candidate markers; thirteen of the proteins have been identified and are therefore of special interest, see Tables 3(a) and 3(b). For representative spectra for the best marker found by Mann-Whitney and ICA, respectively, see Figures 1 and 2. Scatterplots of peak intensities of these two peaks can be seen in Figure 3.

## 4. Discussion

In this exploratory study we aimed to discover potential biomarkers that could distinguish between AD and VaD. Since it is important in an exploratory discovery design to avoid a type II error, we employed two different methods for feature selection [11–13] in order to obtain a more complete list of candidate markers.

In the literature other biomarkers indicating differences between AD and VaD have been described, such as albumin index [14] and the CSF/Serum folate ratio, where a reduced folate ratio was found to be a characteristic of dementia with vascular component [10].

In our sample set, there were statistically significant differences in CSF levels of amyloid *β*1–42, total tau and phosphorylated tau between samples from patients with VaD and AD. However, several recently published large studies report a substantial overlap between the levels in the VaD and AD groups [15].

We found a total of 27 candidate markers, thirteen of which have been identified. We found both up- and downregulated proteins in patients with VaD compared to AD even if the CSF protein concentration was significantly higher in the VaD group. This could be explained by the fact that we did find elevated levels of albumin, IgG, and transferrin, and these proteins comprise approximately 80% of the CSF proteome [16].

Several of the markers found in this study were recognized as markers that we have previously described in studies of AD biomarkers and markers for progression to AD from mild cognitive impairment (MCI) [17–19].

In our previous study we found decreased levels of the CT fragment of Alpha-1-antichymotrypsin in AD compared to healthy subjects [17]. Here we also found decreased levels of the same fragment in the CSF of patients with VaD compared to patients with AD. Thus it seems that levels of this protease inhibitor are lowest in patients with VaD, followed by patients with AD and highest in healthy controls.

We have previously found increased levels of the C-terminal fragment of integral membrane 2B in the CSF of AD and FTD patients compared to healthy controls [18], increased levels of the full length protein have also been described previously [20].

Integral membrane 2B is also known as a BRI peptide, which is known to form insoluble deposits in the brains of patients with familial British and familial Danish dementia [21]. In this study we report reduced levels of the C-terminal fragment of integral membrane 2B in the CSF of patients with VaD compared to AD. Hence, the levels of this protein are similar in patients with VaD and healthy controls but can differentiate between patients with VaD and AD.

In our earlier studies we found increased levels of the C3a peptide lacking the C-terminal arginine in the CSF of patients with AD compared to healthy controls and in patients with MCI who progressed to AD [17–19]. C3a is part of the complement system and involved in inflammatory processes. Here we show lower levels in the CSF of patients with VaD compared to patients with AD. This means that the levels of C3a des-arg in VaD are probably similar to the levels in healthy controls. Hence this marker can differentiate between VaD and AD but not between VaD and healthy aging.

We have previously reported decreased levels of the protease inhibitor Cystatin C in patients with AD compared to healthy controls [17, 18]. In this study we find that the levels of Cystatin C are higher in patients with VaD than in patients with AD. We postulate that the levels of Cystatin C are lowest in patients with VaD, followed by patients with AD, and highest in controls.

We have previously detected increased ubiquitin CSF levels in patients with MCI who progressed to AD [19]. Increased CSF ubiquitin levels have previously been found in both AD and VaD patients [22]. Here we show that VaD patients had significantly lower CSF concentrations of an ubiquitin fragment lacking three C-terminal amino acids compared to AD patients.

Neuroendocrine protein 7B2, also known as Secretogranin V is a molecular chaperone. To our knowledge it has not been previously described in CSF samples from patients with AD or VaD. Mattson et al. described increased levels of Neuroendocrine protein 7B2 in the CSF of patients with frontotemporal dementia (FTD) with high expression of neurofilament [23] In this study we found decreased levels of this peptide in the CSF of VaD patients compared to AD patients.

ApoA-I levels in CSF have previously been found to be decreased in the CSF of AD patients when compared to healthy controls [24, 25]; in this study we also find decreased levels in AD when comparing CSF from patients with AD to patients with VaD.

In addition, we found increased levels of the dimer of ApoA-II in the CSF of patients with VaD compared to patients with AD. In the literature increased levels of monomeric ApoA-II have been found in the CSF of patients with AD compared to healthy controls [20, 26]. However, we were not able to detect the monomeric form of ApoA-II in our assay.

Higher levels of the complement pathway protein C4a des-arg have been described previously in the CSF of patients with AD [20, 26], and our group also found increased levels of the C4a peptide lacking the C-terminal arginine in MCI patients who progressed to AD [19]. Here we find higher levels of the C4a des-arg peptide in the CSF of patients with AD compared to VaD.

Several studies have described albumin as being significantly up- or downregulated in the CSF of AD patients compared to healthy controls, see [27] for review. Here we found albumin to be lower in patients with AD compared to patients with VaD confirming the clinically obtained data on albumin ratios (Table 1). We also found lower levels of IgG in the CSF of patients with AD compared to VaD. These two proteins are used clinically to evaluate blood-brain barrier impairment [28, 29], which is usually more impaired in VaD than AD and consistent with higher Albumin and IgG levels in the CSF of VaD patients.

Furthermore, we found lower levels of transferrin in AD that could perhaps similarly be explained by blood-brain barrier impairment in patients with VaD.

Conflicting results of the CSF levels of retinol binding protein in patients with AD have been described. Increased levels were reported in [20, 24] and in MCI patients who progressed to AD [19] but decreased levels were found in [25]. In the present study we found lower levels of Retinol binding protein in the CSF of patients with VaD compared to AD.

Presently, the occurrence of ischemic changes on computed axial tomography (CAT) scan is a very helpful tool to differentiate between AD and VaD patients. However, we believe that the candidate biomarkers found in this study could aid further in this differentiation.

We report both increased and decreased levels of several proteins and peptides when comparing the CSF from patients with VaD with the CSF from patients with AD. These markers represent various physiological processes such as protein degradation (ubiquitin), protease inhibition (Cystatin C and Alpha-1-antichymotrypsin), and inflammation (C3a and C4a) that are known to be represented in neurodegenerative diseases.

In summary, we found a total of 27 candidate markers for the diagnostic differentiation between VaD and AD. Even for the markers with the lowest *P* values there was considerable overlap between the two groups. Therefore we do not expect any of these markers to be used on its own but in combination with other markers such as the folate ratio, levels of Amyloid Beta 1–42, total Tau, and phosphorylated Tau. All of the thirteen markers identified in this study have been described previously in the literature in the CSF of patients with AD and MCI. To our knowledge, this is the first study where they are described in patients with VaD.

A limitation of the present study is the lack of a healthy control group. Future studies should enroll pathology confirmed AD and VaD dementia cases as well as longitudinally followed control individuals. Furthermore the diagnostic performance of selected candidate markers from this study should be investigated in large prospective multicenter studies.

Despite the obvious application of these novel biomarkers to improve the accuracy of early diagnosis they could also play a role in the development of new disease modifying drugs. This and future proteomic and genomic explorations of clinical samples will likely play an important role in unraveling the biochemistry of dementia pathogenesis ultimately leading to new therapeutic targets.

## Figures and Tables

**Table 1:** Clinical Characteristics; values are means (SD).

Group (no. samples)	Gender M/F	MMSE	Age years	CSF A*β*1-42 pg/mL	CSF total Tau pg/mL	CSF phosphorylated Tau pg/mL	CSF/serum albumin ratio	CSF/serum folate ratio	Total protein g/L
28 probable AD	10/18	20.0 (3.7)	74.6 (11.4)	465 (146)	682 (316)	91 (36)	5.9 (1.7)	2.5 (0.9)	0.38 (0.11)
21 VaD	13/8	21.5 (2.6)	76.2 (8.4)	634 (169)	389 (185)	59 (24)	10.4 (5.5)	1.8 (1.2)	0.62 (0.33)
Mann-Whitney *P* value		n.s.	n.s.	0.011	0.0007	0.0021	0.0005	0.0348	0.002

**Table 2:** Number of peaks and number of significant peaks on the three array surfaces and the two mass ranges used.

Array type	CM10		IMAC30-Ni		H50	
	Peaks	Significant peaks	Peaks	Significant peaks	Peaks	Significant peaks
2–20 kDa	78	38	50	5	38	3
20–200 kDa	55	19	32	2	45	4

**Table:** (a) Univariate analysis—Mann-Whitney

m/z	Array type	*P* value	Direction of change in AD	ID
8295	CM10	0,0017	↑	Ubiquitin −3aa from CT
101801	CM10	0,0075	↓	
4352	CM10	0,0142	↑	Alpha-1-antichymotrypsin CT fragment
4745	CM10	0,0142	↑	
66676	CM10	0,0142	↓	Albumin
3515	CM10	0,0181	↑	Neuroendocrine protein 7B2 CT fragment
17379	CM10	0,0181	↓	ApoA-II dimer
2840	CM10	0,0186	↑	
8614	CM10	0,0274	↑	C4a des-arg
4469	CM10	0,0305	↑	
2430	CM10	0,0387	↑	Integral Membrane 2B CT fragment

**Table:** (b) Multivariate analysis—ICA

m/z	Array type	*P* value	Direction of change in AD	ID
101801	CM10	0,0067	↓	
13105	CM10	0,0067	↑	
147523	CM10	0,0067	↓	IgG
16207	CM10	0,0067	↓	
17379	CM10	0,0067	↓	Apolipoprotein A-II dimer
28058	CM10	0,0067	↓	Apolipoprotein A-I
66676	CM10	0,0067	↓	Albumin
79578	CM10	0,0067	↓	Transferrin
8936	CM10	0,0067	↑	C3a des-arg
90294	CM10	0,0067	↓	
20934	CM10	0,0072	↑	Retinol binding protein
4745	CM10	0,0072	↑	
51402	CM10	0,0077	↓	
3515	CM10	0,0104	↑	Neuroendocrine protein 7B2 CT fragment
5262	IM30Ni	0,01076	↓	
6973	CM10	0,0159	↑	
73422	CM10	0,0182	↑	
2840	CM10	0,0255	↑	
15124	CM10	0,0347	↓	
2249	CM10	0,0467	↓	
13358	CM10	0,0471	↑	Cystatin C
